# The Effect of *Myristica fragrans* on Texture Properties and Shelf-Life of Innovative Chewable Gel Tablets

**DOI:** 10.3390/pharmaceutics13020238

**Published:** 2021-02-09

**Authors:** Inga Matulyte, Akvile Mataraite, Saule Velziene, Jurga Bernatoniene

**Affiliations:** 1 Department of Drug Technology and Social Pharmacy, Lithuanian University of Health Sciences, LT-50161 Kaunas, Lithuania; inga.matulyte@lsmu.lt (I.M.); saule.velziene@lsmuni.lt (S.V.); 2 Institute of Pharmaceutical Technologies, Lithuanian University of Health Sciences, LT-50161 Kaunas, Lithuania; 3 Faculty of Medicine, Lithuanian University of Health Sciences, LT-44307 Kaunas, Lithuania; akvimata0128@kmu.lt

**Keywords:** chewable gel tablet, nutmeg essential oil, gel, mold, firmness, springiness, gelatin, preservative, pharmaceutical research

## Abstract

Chewable gel tablets are an underdeveloped subject, even though there are many simple chewable tablets and gummy candies in the food and pharmaceutical industries. Chewable gel tablets are not as sweet, they can have an active substance, pharmacological effect, and a value of nutrition. The aim of this study was to prepare gelatin-based chewable tablets with *Myristica fragrans* as a preservative and to determine the shelf-life variability depending on storage conditions, and to evaluate texture changes. Firmness and springiness of gel tablets were measured by a texture analyzer and compared between different storage conditions and the shelf-life of tablets was established by mold growing time. Chewable gel tablets were prepared by using silicone form. Mold was most likely to grow on tablets that have been packaged in squeezable bags (after 14 days 60% of all formulations had a mold, *p* < 0.05). The most stable tablets (over 180 days) were in sealed boxes and contained nutmeg essential oil or its solution, or ethanolic nutmeg extract. The gel tablets’ firmness increased about 4 times when they were stored in opened plastic boxes and their springiness decreased about 1.65 times after 28 days in the mentioned conditions, *p* < 0.05. Nutmeg hydrolat had the highest influence on texture variation (*p* < 0.05).

## 1. Introduction

Chewable tablets are a popular dosage form for children, elderly patients or for those who have swallowing disorders. Water is not required for the use of these tablets because they are chewed and saliva moisturizes the tablet mass, which is then easily swallowed, so it is comfortable to use on a journey or in places where it is difficult to get water [1,2]. The most popular chewable tablets are tablets such as paracetamol, acetylsalicylic acid, ibuprofen [3] or other tablets which have an active substance and many excipients, and are mostly opaque or white in color, and are prepared by compression. Another group of chewable tablets are chewable gel tablets which are commonly referred to as gummies [4,5]. These tablets are prepared by using forms (silicone, metal or other) and are not compressed by tablet machines’ punches such as simple (uncoated, coated, soluble or chewable) tablets which are mentioned in the European Pharmacopoeia monograph of Tablets [2,5,6].

Chewable tablets have a pleasant taste and leave no bitterness or an unpleasant taste after eaten. They have many advantages compared to another pharmaceutical forms: they are easy to administrate, water is unnecessary, teaspoons or other cutlery are not required; therapeutic effect of an active substance can be terminated by discarding it before complete ingestion, and they can be used for systemic drug delivery [2,7].

Gel tablets are prepared by using excipients which have gelling properties (gelatin, pectin, tragacanth, starch, agar, and many others). Most gelling agents are polysaccharides and polymers which form gels [2,8,9]. Gelatin is a natural material made from pork skin, beef hide or bones, and its extraction and consumption are growing every year (from 130 tons in 1974 to 305 tons in 2005) [10]. Gelatin made from fish is an alternative to pescatarians though its cost is 4–5-times higher than porcine or bovine gelatin [11]. All gelatin types have many amino acids, approximately 90% protein, which is a good food supplement [10,11]. Gelatin is widely used not only in the food industry but also in the pharmaceutical industry for the making of gelatin capsules and gelatin-based plasma substitutes. Additionally, it is used as an emulsifier, a microencapsulating agent or a stabilizer in some vaccines, and as a gelling agent [10,12,13]. To prepare a biopolymer (gelatin) solution, the use of water is required but it is a good media for microorganisms. There are several methods to prepare a gelatin-based solution but the most popular is to pour cold water on gelatin and leave it for several minutes to swell and then heat it to dissolve the gelatin. Gelatin forms colloidal solutions with water and gels of varying stiffness are produced depending on the concentration [4,5,10,12,14].

Chewable tablets have a sweetener that ensures a good taste. In the food industry there are many sweeteners (natural, semi-natural or chemical). Each sweetener has its advantages and disadvantages, such as sucrose which is a natural sugar produced from sugar beets or sugar canes. It is easily absorbed and provides energy [1,4,15,16]. A huge amount of sucrose in jelly can crystallize and form a layer of sugar on top. To avoid this, polyols are used (sorbitol, glycerol or others), they do not let the sugar crystals to form [1,17]. Artificial sweeteners, although some are sweeter than sucrose, cannot deceive the brain and an organism understands what type of calories were gained and which hormones have to be released from the cells in order to break down the consumed sweeteners. These artificial sweeteners can lead to seizures, headaches, and attention deficit disorders (aspartame as an example) [18].

To manufacture palatable chewable tablets and for them to stand out in the market, sweeteners are not the only substances that should be used as a taste agent; flavorings may be used as well to refer to a specific combined sensation of taste and smell [4,7]. Natural flavors that could be used are herbs, spices, bee products, their extracts, syrups or other forms [19]. They do not only have flavor or taste (honey, vanilla, thyme) but can also create a specific color (acai berry, blueberry, orange) [4,15,20].

Chewable gel tablets have water in their composition which is a good media for bacteria and to prevent the growth of bacteria, it is important to use huge amounts of sweeteners or preservatives. The sweeteners’ amount substitutes water (however, it is not always sufficient as a preservative). Nowadays, it is a trend to consume less sugars, so if their amount is reduced, other materials have to replace them. The preservatives such as citric acid reduce pH values and create an undesirable media for the growth of microorganisms. Other preservatives can kill the microorganisms and prolong a product’s shelf-life [21]. Citric acid and apple acid are the most commonly used to adjust the pH in food products, and sodium benzoate or potassium sorbate are usually used as preservatives, but now there is a new trend to use natural preservatives such as spices and fruit peels [4,22,23,24].

Additionally, microbial contamination is possible due to the contamination of the equipment and the environment. A microbial contamination can do damage to products (reduce shelf-life, change appearance, color, taste). Pharmaceutical forms (solid, semi-solid or liquid) are prone to microbial spoilage or degradation. Any improper packaging and consumption only increase contamination [25]. To protect products from contamination it is necessary to use cleaned devices and to follow good manufacturing practice rules [26,27].

Nutmeg has antioxidant and antimicrobial properties which can be used to protect chewable gel tablets from mold (fungal growth). It has an effect on *Bacillus subtilis*, *Staphylococcus aureus*, *Shigella dysteriae*, *Aspergillus niger*, also, *Myristica fragrans* extracts prepared in different conditions have many other activities against other bacteria [22,28,29,30,31]. Additionally, it was determined that nutmeg essential oil has effect on suppressed *Enterococcus faecalis*, *Streptococcus mutans* (referent), and *Pasteurella multocida* bacteria [32]. Talking about *Enterococcus faecalis*, it is related to oral diseases, such as caries, endodontic infections, periodontitis, and peri-implantitis [33,34]. Using natural essential oil or other materials from nature can help reduce bacterial growth. Antioxidant compounds in nutmeg seeds are: myristphenone, phenolic volatile compounds, phenolic acid (caffeic acid), flavanols (catechin). Compared with other spices and herbs, nutmeg has more than 50% activity of inhibition of DPPH (2,2-diphenyl-1-picrylhydrazyl) [22,35]. Nutmeg can not only preserve a product and prolong its shelf life (which is very important for chewable gel tablets that have water in their composition) but also it decreases bacterial growth in mouth and binds free radicals, this adds additional benefits.

Chewable gel tablets with a natural preservative (such as *Myristica fragrans*) are an innovative pharmaceutical form which can be used as a food supplement or a medical device, and it can have less sugar or sweetness because the natural preservative can prolong shelf-life of gel tablets and it can also substitute the chemical preservative. Nowadays there is a trend of “less sugar” or “natural products”, “from nature/together with nature”, so natural plant as a preservative is a novelty, moreover, it also has some pharmacological effects (antioxidant, anti-inflammatory, antibacterial), and it is a new area in the manufacture and texture analysis of property differences of chewable gel tablets.

The aim was to develop chewable gelatin-based tablets with natural materials used for taste and flavor and to compare the influence of *Myristica fragrans* seed extracts, essential oil, and hydrolat as preservatives to prolong tablets’ shelf life according to the time of onset of mold on the tablets, and to compare the influence of storage conditions on the texture of gel tablets.

## 2. Materials and Methods

### 2.1. Materials

Chewable gel tablets basis was prepared using gelatin (Carl Roth GmbH + Co. KG, Karlsruhe, Germany), distilled water (LUHS laboratory, Kaunas, Lithuania), and glycerol (99+% vegetable origin; Chem-Lab, Zedelgem, Belgium). Thyme herb (A. Karvelis therapy-phytotherapy company, Švenčionys, Lithuania) and sugar (Pfeifer and Langen Marketing Sp., Poznan, Poland) were used for flavor, color, and odor. Sodium benzoate (Berchem, city, Lithuania), 96% ethanol (Vilniaus degtinė, Vilnius, Lithuania), citric acid (Carl Roth GmbH + Co. KG, Karlsruhe, Germany), and *Myristica fragrans* seed (from Grenada; supplier Spaisvilė, Pašaltuonys, Lithuania) extracts, hydrolat and essential oil were used as preservatives. Chewable gel tablets were prepared using silicone gummy bear form. Different concentrations of ethanol were used as an additive material for extracts.

All nutmeg seed products and thyme syrup were prepared in Lithuanian University of Health Sciences, Department of Drug Technology and Social Pharmacy.

### 2.2. Thyme Syrup Preparation

Thyme extract was prepared from thyme herbs and 20% ethanol by using an ultrasound bath. After 30 min, the extract was filtered and was left to settle down. Ethanolic extract was gained from the material in a ratio of 1:1. Thyme extract (three parts) was mixed with 64% of sugar syrup (17 parts) and stored in a refrigerator.

### 2.3. Nutmeg Seed Products Preparation

Nutmeg seed extracts were prepared as follows: one part of nutmeg seed powder was put into the flask and 20 parts of water or 70% ethanol (20 parts) were poured in. The flask with these contents was inserted into an ultrasound bath, after 30 min. the extracts were filtered and poured in a bottle, and stored in a refrigerator.

Essential oil from *Myristica fragrans* seeds was prepared by using a modified Clevenger-type apparatus [36]. Fifteen grams of nutmeg powder and 300 mL of distilled water were used for the preparation of the essential oil. The hydrodistillation process took four hours. Hydrolat was collected as a by-product. Both samples were stored in a refrigerator, in a dark glass bottle.

### 2.4. Chewable Gel Tablets’ Preparation and Compositions

The pilot-study was performed with different amounts of gelatin, water and thyme syrup. Five different compositions were prepared and one base of chewable tablets was selected based on visual and organoleptic methods.

The samples of gel tablets were prepared as follows: gelatin was mixed with water and glycerol solution, and stored for 10–15 minutes to swell. After swelling, the gelatin solution was heated in a water bath (60 °C). Thyme syrup (room temperature) was added in the gelatin solution and mixed, then the preservative was added. The chewable gel tablets’ mass was poured in silicone forms and left for 24 hours to solidify (20 ± 5 °C). Gel tablets were removed from the forms and further investigations were carried out. The gel tablets’ compositions are presented in Table 1.

The gel tablets of each series were stored in five different locations: in plastic boxes in a refrigerator at 8 °C (PBR) and in a freezer at −15 °C (PBF), in a squeezable plastic bag (SR) and on an opened plastic pallet (OR), and in a closed plastic box (CR) at room temperature (20 ± 5 °C).

### 2.5. Chewable Gel Tablets’ Physical Parameters: Mass Change, Firmness and Springiness

Throughout the study, the mass of gummies was measured and the mass loss with mass variation were calculated for all series of gel tablets. Chewable gel tablets were retrieved from the freezer and kept at room temperature for 30 min and then weighed. The influence of the storage conditions on the weight of the gel tablets was determined.

Chewable gel tablets’ firmness and springiness were measured by texture analyzer TA.XT.plus (Texture Technologies, Brewster, NY, USA). The “Gummy confectionery” test was chosen. Parameters of the test: return speed 10 mm/s; force 1 g; strain 50%; pre-test 1.00 mm/s test speed 1.00 mm/s; post-test speed 10 mm/s; hold time 60 s; and trigger force 5.0 g. Changes of texture of the chewable gel tablets were analyzed after one day and one month for the OR series gel tablets.

### 2.6. Chewable Gel Tablets’ Quality Determination

The quality of the gel tablets was assessed by the appearance of mold (assayed fungal growth during storage) [15]. The longer the gel tablets do not mold, the more effectively the preservative acts against microorganisms. The influence of preservatives on the quality of the gel tablets was determined during the stability study: the days after which mold began to grow on the chewable gel tablets in a plastic box (CR) or the ones stored in the refrigerator (PBR) were counted.

### 2.7. Microbial Contamination of Equipment

Microbial species were selected according to a Pharmacopoeia Article (5.1.4., Microbiological quality of non-sterile pharmaceutical preparations and substances for pharmaceutical use—“Special Ph. Eur. Provision for oral dosage forms containing raw materials of natural (animal, vegetal or mineral) origin for which antimicrobial pretreatment is not feasible and for which the competent authority accepts TAMC (total aerobic microbial count) of the raw material exceeding 10^3^ CFU/g or CFU/mL”) and the swab samples were taken from the equipment and containers. All tests of microbial growth were performed according to European Pharmacopoeia methods [37,38].

Before research, tools and equipment were cleaned with 96% ethanol, work was done with gloves. Chewable gel tablets were manufactured and tested according to the rules of good manufacturing practice.

### 2.8. Statistical Analysis

Data are presented as the mean ± SD. Statistical analysis was performed using Student’s *t* test. The results were significant when *p* < 0.05.

## 3. Results and Discussion

### 3.1. Chewable Gel Tablets’ Composition

A pilot-study was performed and the tablet base was selected before starting the study on the influence of nutmeg as a preservative on the quality of chewable tablets. The primary mass of chewable tablets with different gelatin concentrations (five compositions) was prepared and the G0 composition (Table 1) was selected according to the best physical parameters, taste and sensation, and a study was initiated with different preservatives. Gelatin concentration in five bases was from 7.69% to 15.38% and the best chewable gel tablets’ base was determined with 13.46% of gelatin. In the Morten et al. (2018) study on gelatin-based pharmaceutical oral formulation, 8.8–10% concentration of gelatin was used [39]. Concentrations of gelatin can vary greatly depending on which gelatin is selected, there are many categories of gelatin which can be used in food or pharmaceutical industry [10]. Gelatin of different hardening strength, purity and bloom number is used in different studies. To prepare gummy candies, the Pizzoni et al. (2015) study of gummy candies with strawberry flavors used 6.5% of gelatin, and the Deleris et al. (2011) study about candies and aroma release dynamic used 0–15% of gelatin [40,41]. Chewable gel tablets are similar to gummies or candies, but they differ from them—they are eaten not as a desert but as a food supplement or a medical substance, and they have a medical and nutrition purpose because they are equivalent to chewable tablets which have a recognition in the European Pharmacopoeia [6].

After evaluating the composition of G0, it was observed that after some time the sugar on the surface of the chewable tablets begins to crystallize, therefore glycerol was added to the composition. Glycerol and other polyols are used as additives to protect from sucrose crystallization [2,17,42,43]. In this research 9.61% of glycerol was used, which not only protects from sugar crystallization, but also gives a sweet taste for the tablets [44]. Glycerol also protects chewable tablets from water loss and the mass of tablets does not change a lot when stored in an open package.

**Figure 1:**
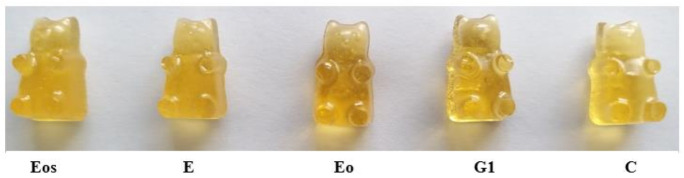
Chewable gel tablets’ (Eos, E, Eo, G1, C) color pallet. The chewable tablets’ composition code is given in Table 1.

All chewable gelatin-based tablets had thyme syrup in composition. Thyme (*Thyme vulgaris* L.) herb has antiseptic and tonic effects and today it is used as a respiratory remedy which can reduce cough. Extracts, essential oil or a tea can be produced from its herb. Thyme gives an acceptable flavor for food, medicine and cosmetics. Chewing tablets with thyme (syrup was prepared from liquid thyme extract) can decrease the amount of microbes in the mouth. In traditional medicine, thyme was used as an antiseptic, antispasmodic, antitussive, antifungal, antioxidative material [45,46]. Thyme was chosen because of its wide pharmacological properties and as a flavor and color corrector in chewable gel tablets. All chewable tablets were yellow (amber) in color and had a pleasant taste apart from the fact that the used preservatives changed the transparency of the tablets (Figure 1).

Three tablets (Eo, G1 and C) of five (in Figure 1) were transparent, the Eo tablet was of the darkest color compared with all other chewable tablets’ composition, which are presented in Figure 1. The Eos and E tablets were opaque, and a difference in the color of the gelatin-based tablets with the use of ethanolic extract or ethanolic essential oil solution was not found. This picture shows how different the colors of gel tablets can be by using a different preservative and its forms. All tablets (10 compositions) were yellow or amber color with a different undertone.

### 3.2. Chewable Gel Tablets’ Weight Variation and Quality

**Figure 2:**
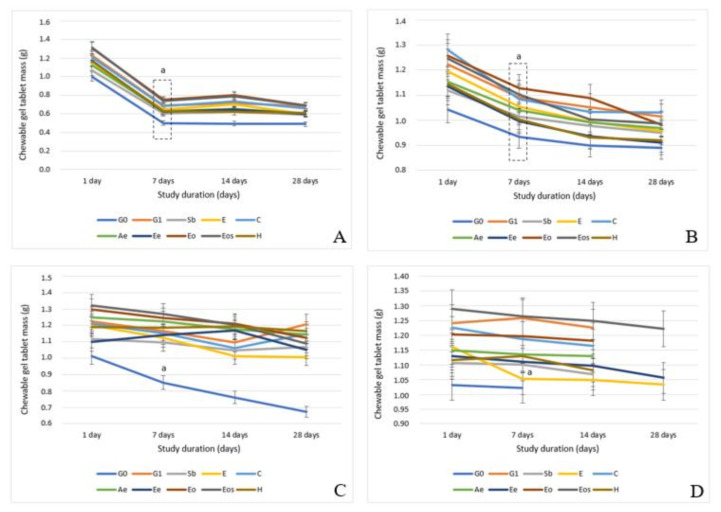
Chewable gel tablets’ mass variation: (**A**) in an open plastic bag at room temperature (OR); (**B**) in a plastic box in a freezer (PBF); (**C**) in a plastic box in a refrigerator (PBR); (**D**) in a squeezable plastic bag at room temperature (BR), the expired curve in (**D**) shows that the tablets became moldy and were not weighed; *n* = 8; a—*p* < 0.05 vs. mass after 1 day.

The mass changes in different storage locations were determined. All chewable gel tablets were weighed for four weeks and the differences of mass are presented in Figure 2. In Figure 2A, the mass variation in a room temperature in an open plastic bag is presented.

All samples’ tablets (Figure 2A) weighed from 1.001 ± 0.054 g to 1.314 ± 0.039 g after one day, after a month (28 days) the tablets’ mass interval was from 0.491 ± 0.027 g to 0.788 ± 0.033 g. Certain storage conditions (temperature 20 ± 5 °C and an opened box) caused the tablets to lose from 44.08% to 50.95% of their weight—the water in the tablets evaporated and the texture changed. In this study, it was observed that the tablets containing glycerol (all except G0) increased in weight after two weeks (this was due to the increased humidity in the room due to changed weather conditions). Glycerol attracted moisture from the environment to the tablets. Based on the study by Ramos et al. (2013), the moisture in the films varies depending on the glycerol concentration (from 17.91% to 21.71%, glycerol concentrations 40–60%) [47].

Moreover, tablets without glycerol had the smallest mass and were harder than gel tablets containing glycerol. Keeping chewable gel tablets in an open bag at room temperature is a wrong decision because they can lose not only water but also their active substances, which are volatile, and the texture of tablets are too hard to be chewed, although after 6 months all of the tablets remained unaffected by a mold.

Chewable gel tablets in a refrigerator (PBR) and a freezer (PBF) were kept in these conditions for one month. Before weighing the tablets, they were taken out from a refrigerator/freezer and left for 30 minutes in a room temperature (in a closed container). Then the tablets were weighed and the results are presented in Figure 2B,C.

Chewable gel tablets from the freezer (PBF, Figure 2B) lost from 15.01% to 21.94% of their mass after 28 days. The highest amount of water and other liquid material was lost from Eo, Eos, Ee, and E samples. All samples had essential oil or ethanol extract which can evaporate together with water therefore they have more changes in mass. The lowest mass variation was determined in G0 and Sb, 14.67% and 15.01%, respectively. The tablets without glycerol from a freezer had the lowest mass variation. It can be concluded that freezing and thawing have the lowest effect on these gel tablets because only water evaporates and glycerol does not attract moisture from the environment since the temperature is too low (water is frozen) and the thawing time is too short for all of the formulations. Glycerol amount in these chewable gel tablets was 9.6%. Based on the literature, 10% glycerol solution’s freezing temperature (a point when the solution freezes) is −1.6 °C [48,49]. In this study the temperature was −15 °C so glycerol also froze and there was no incidence of moisture attraction from the environment.

Comparing the weight loss with the results gained from the gel tablets kept in refrigerator (PBR, Figure 2C), the G0 gel tablets lost over 33% of mass and the other samples lost less than 18% of their weight (tablets which have essential oil or ethanol in their composition lost more than 10% of their content). It was observed that glycerol attracted moisture to the gel tablets, but no correlation was found between the study duration and weight change, some weight variations were determined in 7–14 days interval, others—14–28 days.

The direct effect of preservatives on the changes in tablet weight was not determined, unless the preservative was a volatile compound (essential oil, ethanol)—they evaporated under environmental conditions.

The smallest changes in mass were determined in a squeezable plastic bag (BR, Figure 2D) and in a closed plastic box (CR) at room temperature (this sample had less variation so data are not presented separately).

**Figure 3:**
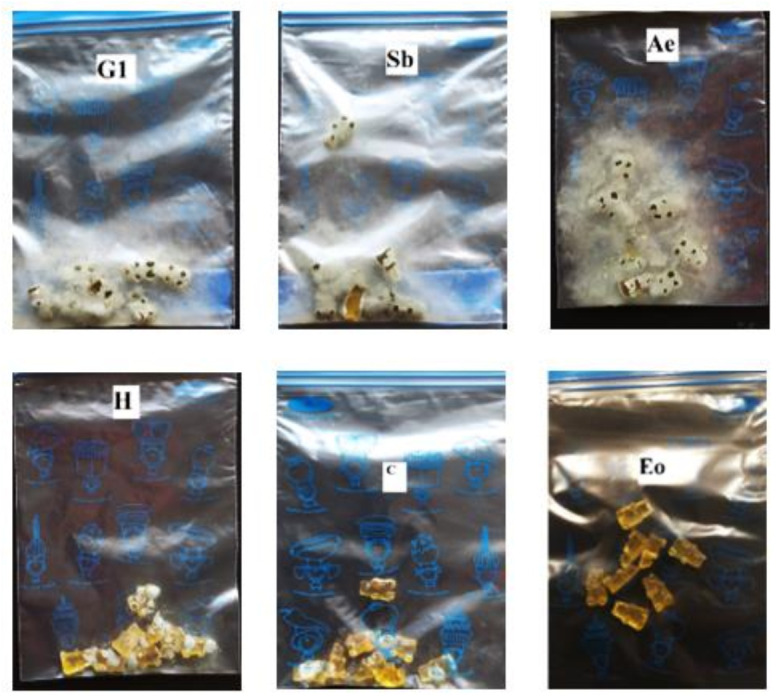
Mold growth on chewable gel tablets (G1, Sb, Ae, H, C, and Eo) after 52 days, *n* = 8 (number of tablets in the plastic bag).

**Figure 4:**
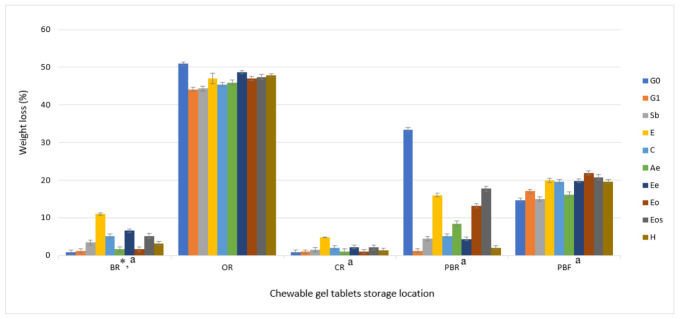
Dependence of changes in the mass of chewable gel tablets on storage conditions, *n* = 8. * the change in mass was calculated from the last results before appearance of mold; a—*p* < 0.05 vs. OR conditions.

**Figure 5:**
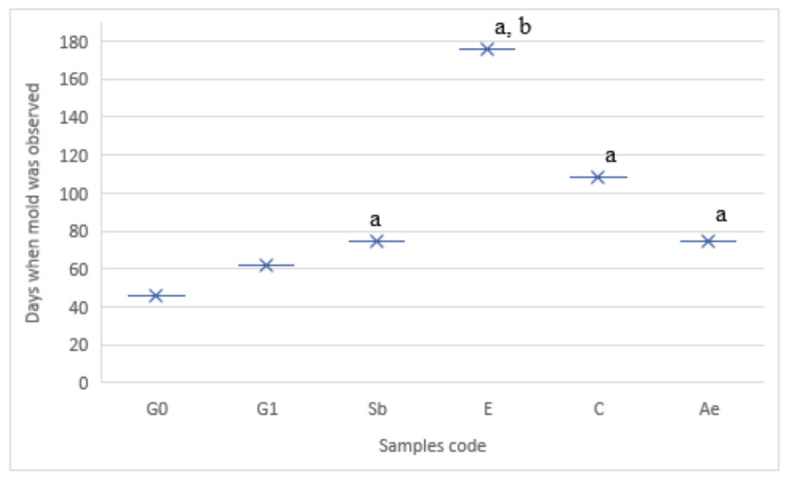
Chewable gel tablets shelf-life in a closed plastic box at room temperature (CR). a—*p* < 0.05 vs. G1; b—*p* < 0.05 vs. C; *n* = 8; * study duration was 180 days; Ee, Eo, and Eos did not have a mold after 180 days, these samples are not presented in this figure.

In a squeezable plastic bag as a storage location, it was determined that the gel tablets in there had the most variable shelf-life. In SR conditions, 70% of the samples developed a mold after 14 days (sample G0 earlier). Only three samples (E, Ee, and Eos, Figure 2D) did not have a mold after 28 days. The Ee and Eos samples were stable for 75 days and the E for 52 days. These results show that ethanol and nutmeg ethanolic extract or nutmeg essential oil ethanolic solution have a higher influence on chewable gel tablets’ shelf-life than sodium benzoate, citric acid or other solutions. These results (SR) were influenced by the chosen packaging (squeezable bag, Figure 3) because results in a plastic box at room temperature (CR) were different. The mass variation (Figure 4) was smallest and the shelf-life was longest in CR conditions (Figure 5).

Eight chewable gel tablets of every composition were kept in separate plastic bags and the number of days until mold had grown on the tablet was determined. When mold was seen on gel tablets in the plastic bag, the study was stopped.

The bacterial contamination tests were completed for equipment, devices and containers (Table 2). The amount of detected microorganisms was not higher than allowed. Less than 1.0 × 10^1^ of TAMC (total aerobic microbial count) and TYMC (total mold and yeast count) were found, other organisms were not found. In the Ratajczak et al. (2014) study it was determined that some of the pharmaceutical products with raw materials or herbal medicinal products have a higher risk of microbial contamination [50]. Therefore, it is important to use good manufacturing practice rules for the development of products.

**Figure 6:**
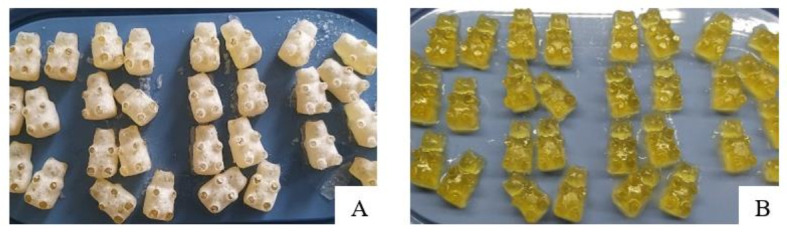
Chewable gel tablets taken out of freezer (**A**) and after 30 min at room temperature (**B**), tablets’ amount per one series is *n* = 8.

To compare storage conditions and containers, the opened plastic bag (OR) had the highest influence on the weight loss of gel tablets, all samples lost over 40% of mass and it was determined that glycerol significantly protects chewable gel tablets from weight loss when the tablets are in an open-air container. Temperature below zero (PBF) has an influence on water separation from gel tablets base—as was determined when they were left in a room temperature for 30 min (Figure 6). The safest containers to hold chewable gel tablets mass’ uniformity is an airtight box (CR). These storage conditions have the smallest influence on weight loss and a higher influence on shelf-life (Figure 3).

In Figure 6, four different formulations of tablets are shown, one formulation has 8 tablets (from each series of each gelatin-based tablets formulations for chosen storage conditions) with which the studies have been performed.

Ethanol and ethanolic nutmeg extracts in a composition of chewable gel tablets (E, Ee, Eos) have a higher influence on mass loss compared to other samples because they evaporate. However, these excipients significantly increased shelf-life of gel tablets (Figure 5).

Chewable gel tablets with nutmeg essential oil or extract had a longer shelf-life than other tablets. Ee, Eo, and Eos samples’ shelf-life was over 180 days. They all had nutmeg as a preservative. Nutmeg essential oil has influence on microorganism growth suppression, 0.2% of essential oil suppressed the growth of *Pasteurella multodica* [32]. The most likely to mold tablets are those that did not contain preservatives and glycerol (G0 and G1). The shelf-life of the tablets that contained sodium benzoate was the same as the tablets containing aqueous nutmeg extract (in Figure 3, gel tablets in a squeezable bag with growing molds are presented). The chewable gel tablets with citric acid had a higher influence on protection from mold growth than sodium benzoate, which is usually used as a preservative in food or pharmacy industries. Sodium benzoate is an artificial preservative which has negative effects on health, it can be dangerous and can cause asthma, cancer, and hypersensivity [51]. It is used in concentrations of 0.02–0.5% in oral medicines [52]. So, this study shows that sodium benzoate (0.096%) can be replaced by aqueous nutmeg extract (1.92%), nutmeg hydrolat (1.92%) or citric acid (0.96%), which have similar or better effects against mold (Figure 5).

Cinnamon, cloves, rosemary extract, grapefruit extract and other spices, herbs or fruits can be used as natural preservatives [4,53]. Thyme extract was chosen as a taste additive but it also has antiseptic and antifungal activities [46]. In the study by Bartkienė at al. (2018), *Thymus vulgaris* essential oil had a higher effect against such microorganisms as *C. paradisi, C. recutita*, and *Eugenia caryophyllata* than other essential oils in gummy candies, such as cloves essential oil. The essential oil’s concentration ranged from 0.1% to 0.4% or an essential oil emulsion with modified waxy maize starch was used, which contained 12% of essential oil [8].

Citric acid is used as an antioxidant, a preservative and a taste coregent in hard candies at a concentration of 0–1% [54] and in gummy candies from 2.5% to 4.5% [55]. In this study, 0.96% citric acid concentration was not only used as a preservative for the chewable gel tablets but also to produce a better taste. Finally, this concentration was more effective than sodium benzoate.

### 3.3. Chewable Gel Tablets’ Firmness and Springiness

**Figure 7:**
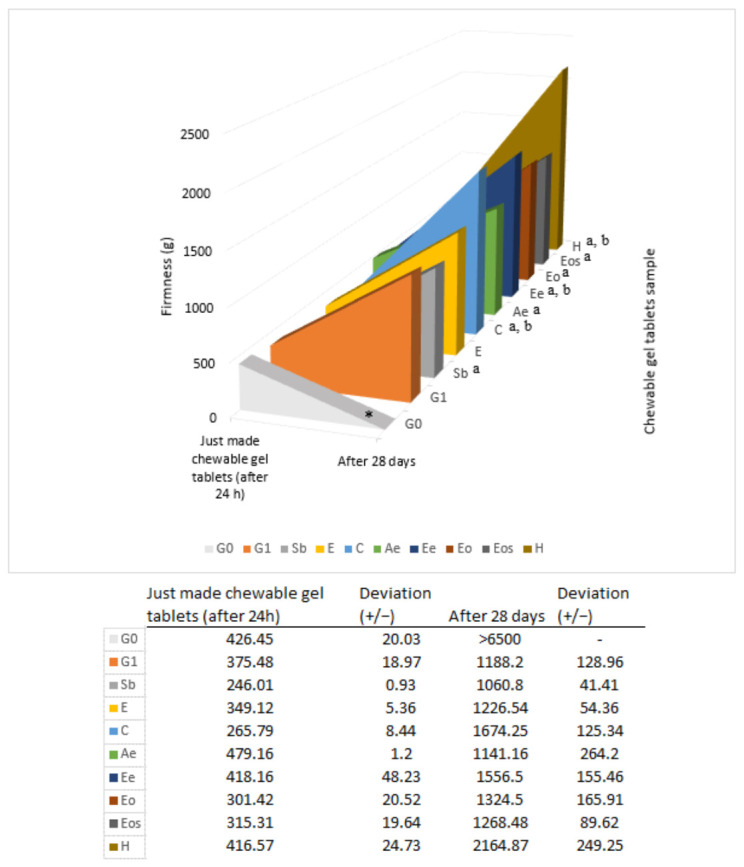
Firmness changes in chewable gel tablets after 28 days kept at room temperature in an open plastic bag. * Device did not measure value after 28 days, it was higher than 6500 g, a statistically significant change after 24 h vs. G1; b—statistically significant change after 28 days vs. G1, *p* < 0.05, *n* = 8.

To determine the preservatives’ influence on the chewable gel tablets’ texture, a study was prepared where the firmness and springiness of the gel tablets were measured in OR storage conditions. Firmness is a parameter which shows chewable gel tablet’s strength and tightness. In this study it was measured how much strength is needed to compress 50% of gel tablet’s height. In Figure 7 the change of firmness is shown. Chewable gel tablets without glycerol (G0) had a higher firmness—from 426.45 ± 20.03 g to over 6500 g (the analyzer did not measure the correct value). After 24 hours, the firmness of the gel tablets with preservatives was measured and it was determined that aqueous extract of nutmeg (Ae), ethanolic extract of nutmeg (Ee), and nutmeg hydrolat statistically significantly increased the firmness by about 16.64 ± 7.76%, but sodium benzoate (Sb), citric acid (C), nutmeg essential oil (Eo), and essential oil solution (Eos) significantly decreased the firmness by about 24.86 ± 9.90% compared to the G1 sample. To compare the preservatives’ influence on firmness after 28 days, it was determined that the force needed to compress the gel tablets by 50% of H, C, and Ee samples was significantly higher than in the G1 sample (2164.87 ± 249.25 g, 1674.25 ± 125.34 g, and 1556.5 ± 155.46 g vs. 1188.2 ± 128.96 g). Other results did not show a statistically significant change after 28 days (Figure 7).

**Figure 8:**
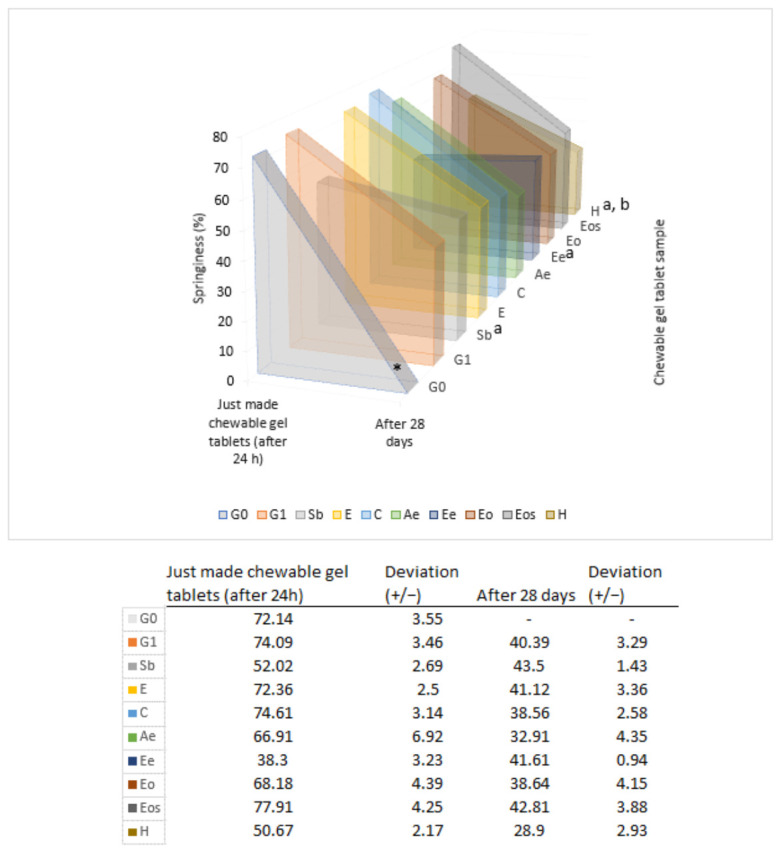
Springiness’ change in chewable gel tablets after 28 days kept at room temperature in an open plastic bag. * Device did not measure value after 28 days, a statistically significant change after 24 h vs. G1; b—statistically significant change after 28 days vs. G1, *p* < 0.05, *n* = 8.

When evaluating springiness (parameter which shows elasticity) in the chewable gel tablets with preservatives, it was determined that nutmeg hydrolat had a statistically significant influence on lower springiness for both just made and 28-day-old gel tablets (31.61% and 28.45% lower to compared with G1). After 24 hours, sodium benzoate (Sb) and a sample with ethanolic nutmeg extract (Ee) also had a statistically significantly decreased springiness from 74.09 ± 3.46% (G1) to 52.02 ± 2.69 and 38.30 ± 3.23% (Figure 8).

Evaluating the results of the chewable gel tablets’ textures, which were achieved with different preservatives, it can be concluded that the tablets’ firmness increase nearly 4 times and springiness decreased by about 1.65 times when kept in such storage conditions as an opened plastic box at room temperature. In the study by Bartkienė et al. (2018), gummy candies’ hardness, gumminess, and chewiness were measured. When thyme or cloves essential oil (0.2%) were added to the gummies, the hardness decreased from about 5400 to 2900 g (thyme) and to 3400 g (cloves), the gumminess decreased by about 1.3 times and the chewiness increased by about 1.14 and 1.08 times [8]. In the study by Čižauskaitė et al. (2019) where gummy bears with vitamin D3 and calcium carbonate were prepared, the texture variation was determined. Statistically significant results of decreasing firmness, strength, and hardness were obtained when active substances were added. The firmness decreased over 50%, hardness—about 30%, strength—about 40% [4].

The texture of the chewable gel tablets is affected not only by storage conditions but also by excipients and active ingredients.

The study with nutmeg seed products as a preservative is the beginning of many studies of the future where natural materials (plants, herbs, seeds) can be used as natural preservatives in solid medicines forms (such as gel tablets which have water in their composition). Additionally, these materials can modify tablets’ texture properties so the results can be used for the manufacture of chewable gel tablets. Another benefit of natural materials from plants is that extracts and essential oils have a pharmacological effect and can be used as synergistic materials together with pharmaceutical substances.

## 4. Conclusions

After the production of 10 different formulations of chewable gel tablets, it was found that their shelf-life was influenced not only by the choice of preservative but also by the storage conditions. The chewable gel tablets are most likely to become molded in squeezable bags, and the most suitable tablets for use were stored in tightly closed boxes. When gel tablets were stored in the freezer and then left at room temperature for 30 min, they lost part of water, which is visible next to the tablets; and when stored in an open plastic box they lost part of their weight and hardened. The best way to store gel tablets is in a sealed box.

Nutmeg essential oil and its solution, ethanolic nutmeg extract were better preservatives for chewable gel tablets than sodium benzoate or citric acid (*p* < 0.05). The shelf-life (in a closed plastic box at room temperature) of these gel tablets (with nutmeg seed product) was more than 62 days longer compared to gel tablets containing citric acid (0.96%) solution.

Nutmeg hydrolat had the biggest influence on texture as a preservative. It increased the gel tablets’ firmness by about 1.8 times and decreased elasticity by about 1.4 times compared to the tablets without preservatives after 28 days of study (*p* < 0.05). The gel tablets without glycerol are incompressible and very hard after 28 days, therefore, glycerol is required as a protector from weight loss.

This study shows that a natural preservative (nutmeg seed products) can substitute chemical preservatives and protect gelatin-based tablets from mold and prolong the shelf-life of tablets. These data are the basis for further research into the use of natural substances (extracts, essential oil) in the protection of medicines or other forms (food supplements) against mold and the additional exploitation of the pharmacological effects of plants.

## Figures and Tables

**Table 1:** Chewable gel tablets’ composition.

Ingredients	Samples Series									
	G0	G1	Sb	E	C	Ae	Ee	Eo	Eos	H
Gelatin (g)	7.0	7.0	7.0	7.0	7.0	7.0	7.0	7.0	7.0	7.0
Distilled water (mL)	20.0	18.0	17.0	17.0	17.0	17.0	17.0	18.0	17.0	17.0
Glycerol (g)	-	5.0	5.0	5.0	5.0	5.0	5.0	5.0	5.0	5.0
Thyme syrup (g)	25.0	22.0	22.0	22.0	22.0	22.0	22.0	22.0	22.0	22.0
Sodium benzoate solution 5% (mL)	-	-	1.0	-	-	-	-	-	-	-
96% ethanol (mL)	-	-	-	1.0	-	-	-	-	-	-
Citric acid 50% solution (mL)	-	-	-	-	1.0	-	-	-	-	-
Aqueous nutmeg extract (mL)	-	-	-	-	-	1.0	-	-	-	-
Ethanol nutmeg extract (mL)	-	-	-	-	-	-	1.0	-	-	-
Nutmeg essential oil (µL)	-	-	-	-	-	-	-	10.0	-	-
Ethanol nutmeg essential oil 10% solution (mL)	-	-	-	-	-	-	-	-	1.0	-
Nutmeg hydrolat (mL)	-	-	-	-	-	-	-	-	-	1.0

**Table 2:** Chewable gel tablets’ composition.

Micro-Organisms	Equipment and Devices (Scales, Texture Analyzer, Tweezers, Silicone Form and etc.)	Containers (Plastic Box) (the Plastic Squeezable Bag Was Not Explored; It Was a Single-Use Plastic Bag, Taken Straight from the Supplier Box (Non-Contaminated))
TAMC	<1.0 × 10^1^	<1.0 × 10^1^
TYMC	<1.0 × 10^1^	<1.0 × 10^1^
Bile-tolerant gram-negative bacteria	not detected	not detected
*Salmonella* spp.	not detected	not detected
*Escherichia coli*	not detected	not detected
*Staphylococcus aureus*	not detected	not detected

## Data Availability

The data presented in this study are available on request from the corresponding author.
